# In-person and online mixed method non-randomised studies exploring feasibility and acceptability of HEADS: UP, an adapted Mindfulness-Based Stress Reduction programme for stroke survivors experiencing symptoms of anxiety and depression

**DOI:** 10.1186/s40814-024-01545-w

**Published:** 2024-09-12

**Authors:** Maggie Lawrence, Bridget Davis, Naomi E. Clark, Jo Booth, Graeme Donald, Nadine Dougall, Madeleine Grealy, Bhautesh Jani, Jennifer MacDonald, Helen Mason, Margaret Maxwell, Ben Parkinson, Matilde Pieri, Xu Wang, Stewart Mercer

**Affiliations:** 1 Research Centre for Health (ReaCH), Glasgow Caledonian University (GCU), Glasgow, Scotland G4 0BA UK; 2 School of Health and Society, University of Salford, Salford, M6 6PU UK; 3 Health and Social Care Sciences, Edinburgh Napier University, Edinburgh, EH11 4BN UK; 4 Psychological Services and Health, University of Strathclyde, Glasgow, G1 1XQ UK; 5 General Practice and Primary Care, School of Health and Wellbeing, MVLS, University of Glasgow, Glasgow, G12 9LJ UK; 6 Yunus Centre, GCU, Glasgow, G4 0BA UK; 7 Nursing, Midwifery and Allied Health Professions Research Unit, Faculty of Health Sciences and Sport, University of Stirling, Stirling, FK9 4LA UK; 8 School of Humanities and Social Sciences, Leeds Beckett University, PD405 Portland Building, City Campus, Leeds, LS1 3HE UK; 9 Primary Care and Multimorbidity, Usher Institute, University of Edinburgh, Edinburgh, Scotland EH8 9AG UK

**Keywords:** Stroke, Anxiety, Depression, Mindfulness, Self-management, Feasibility, Acceptability, Group-based, Online, In-person

## Abstract

**Background:**

Depression and anxiety are prevalent after stroke and associated with poor outcomes. We previously co-developed a stroke-specific self-management intervention, HEADS: UP (Helping Ease Anxiety and Depression after Stroke). The two studies reported here aimed to test the feasibility and acceptability of the HEADS: UP course and supporting materials, and research processes ahead of a definitive trial.

**Methods:**

We recruited community-dwelling stroke survivors (SS) ≥ 3 months post-stroke, with symptoms of mood disorder (Hospital Anxiety and Depression Scale ≥ 8). Participants could ‘enrol’ a family member/ ‘other’ to take part with them, if desired. Study 1 tested HEADS: UP delivered in-person, and informed optimisation of research processes and intervention delivery and materials. In a pragmatic response to Covid-related socialising restrictions, HEADS: UP was then adapted for online delivery, tested in Study 2. The primary outcome (both studies) was the feasibility (acceptability, fidelity) of the intervention and of research processes. Quantitative data (including patient-reported outcome measures (PROMs) assessing mood and quality of life) and qualitative data were collected pre-/post-intervention. Descriptive statistics were used to analyse quantitative data; a thematic framework approach was used to analyse qualitative data. Both studies received ethical approval prior to commencement.

**Results:**

**Study 1**

Feasibility: 13 (59.1%) of 22 potentially eligible stroke survivors consented; aged 66 (median, interquartile range (IQR) 14); male (*n* = 9; 69%); 28 (IQR 34) months post-stroke. Of these, *n* = 10 (76.9%) completed PROMS pre-intervention; *n* = 6 (46.2%) post-intervention.

Acceptability: Nine (69.2%) of the 13 participants attended ≥ 4 core intervention sessions. Aspects of screening and data collection were found to be burdensome.

**Study 2**

Feasibility: SS *n* = 9 (41%) of 22 potentially eligible stroke survivors consented; aged 58 years (median; IQR 12); male (*n* = 4; 44.4%); 23 (IQR 34) months post-stroke. Of these, *n* = 5 (55.6%) completed PROMS pre-intervention; *n* = 5 (55.6%) post-intervention.

Acceptability: Five (55.6%) of the 9 participants attended ≥ 4 core sessions. They found online screening and data collection processes straightforward.

**Supplementary Information:**

The online version contains supplementary material available at 10.1186/s40814-024-01545-w.

## Key messages regarding feasibility

### 1) What uncertainties existed regarding the feasibility?

The feasibility of using a community-based recruitment strategy to recruit community-dwelling stroke survivors to HEADS: UP, a stroke-specific Mindfulness-Based Stress Reduction course, in-person or online, and their adherence to the course and to the research processes were uncertain because this intervention had not been tested with people affected by stroke.

### 2) What are the key feasibility findings?

Recruitment was feasible but further development of the community-based recruitment strategy is required to boost equality, diversity and inclusion of participants. The intervention was implemented as intended and was valued by participants for its impact on symptoms of anxiety, depression and quality of life.

### 3) What are the implications of the feasibility findings for the design of the main study?

HEADS: UP proved feasible and acceptable when delivered in-person or online. In future work, we will explore strategies to optimise recruitment, including equality, diversity and inclusion.

## Background

Stroke, a common chronic disabling condition, affects more than 80 million people globally [1]. It is estimated that, despite many recent developments that reduce mortality rates and symptom severity, there are 13.7 million new stroke events annually [1]. The effects of stroke are many and varied and may include physical, communication and cognitive impairments, as well as mood disorder — predominantly depression and anxiety [2]. Depression and anxiety are prevalent after stroke, affecting one in three people in the first year [3]. Symptoms persist long-term [4, 5] and are associated with poor outcomes, including lack of engagement with rehabilitation and recovery [6, 7]. Long-term psychological support is a recognised unmet need [8] and a research priority [8, 9]. Innovative, community-based self-management interventions have the potential to meet individual needs and address this gap in service provision.

Mindfulness-Based Stress Reduction (MBSR) is a widely used, structured, 8-week course [10]. Daily practice, sustained over time, is an essential element of engagement and improved outcomes. MBSR is effective in the treatment of anxiety and depression in diverse clinical populations including multiple sclerosis and Parkinson’s disease [11, 12] and in a range of settings [13]. Following stroke, systematic review evidence indicates MBSR may be beneficial across a range of outcomes including depression and anxiety [14]. A recent scoping review found mixed evidence regarding mindfulness interventions as effective psychological interventions post-stroke and indicated that further research is warranted [15].

**Fig. 1:**
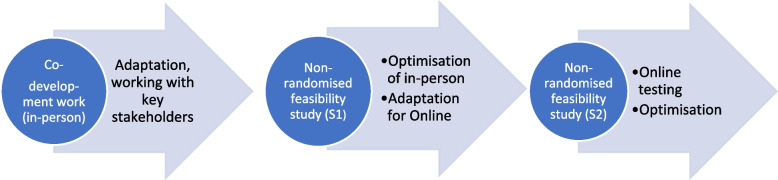
HEADS: UP programme of research: adaption, optimisation, testing

As part of a programme of complex intervention research [16, 17] we worked with stroke survivors and family members to co-develop HEADS: UP (Helping Ease Anxiety and Depression after Stroke), a stroke-specific adaption of a standard group-based MBSR course [18]. This paper reports two non-randomised feasibility studies undertaken subsequently, and ahead of a definitive trial (see Fig. 1).

## Methods

The two non-randomised studies (Study 1, in-person delivery; Study 2 online delivery) aimed to test feasibility and acceptability of the HEADS: UP intervention (course and supporting materials) and research processes from the perspectives of stroke survivors and family members ahead of a planned pilot RCT. Both studies aimed to answer the broad question: what elements of intervention design, delivery and study processes require to be optimised ahead of an RCT? Study objectives focused on determining feasibility and acceptability of recruitment, retention, adherence, outcome measures and the intervention itself, assessed using mixed methods [16, 19–21]. Reporting is guided by recommendations from Lancaster and Thabane (2019) [22] and based on the CONSORT extension for randomised pilot and feasibility trials [20] (see Additional file 1).

Study 1 was undertaken in-person, in accordance with the original programme design. Analysis of the study data informed subsequent optimisation of the HEADS: UP intervention and research processes. Sudden imposition of UK Covid-19 restrictions, March 2020, caused us to alter the planned research programme. Consequently, following optimisation of the in-person intervention and research processes, rather than conducting a pilot RCT, we adopted a pragmatic approach and, having secured agreement from the funder, adapted HEADS: UP for online delivery. We then tested HEADS: UP Online (intervention; research processes) in a second non-randomised feasibility study, Study 2 (see Fig. 1).

### Study 1: HEADS: UP in-person delivery

The HEADS: UP course comprises nine weekly sessions, i.e. an Introductory session and eight core sessions; attendance at ≥ 4 of the 8 core sessions was used as an indicator of feasibility. Sessions last approximately 2.5 h, including a 30-min break, except in week 7 when a 6-h silent retreat is offered. HEADS: UP was delivered in December 2019 in an accessible venue on a city-centre university campus. We offered to pay transport costs or organise taxis for participants, as required. Refreshments were available on arrival and during the break. We provided course materials including chapters from the course manual in weekly instalments (paper/electronic according to preference), audio recordings of practices which complemented the sessions (CDs or downloadable electronic files) and equipment such as yoga mats and foam blocks. HEADS: UP was co-delivered by two experienced MBSR trainers who adhered to contemporaneous Good Practice﻿ Guidelines [23] and had previously undertaken bespoke HEADS: UP Train the Trainer (TtT) training to enhance stroke awareness and competence. We provided trainers with logbooks to record any divergence from the HEADS: UP manual and comments that might enhance future iterations of course delivery.

To promote intervention adherence, we planned to deliver HEADS: UP to dyads, i.e. a stroke survivor plus a family member or other person of the individual’s choosing. Hereon in, the latter is referred to as ‘family member’. As the planned unit of analysis for the outcome measures was the stroke survivors, in this paper, the term ‘participants’ refers to the stroke survivors. From an estimated attrition rate of approximately 23% [14], we aimed to recruit 14 community-dwelling dyads who were able/willing to travel to the venue; however, we did not exclude potential participants who did not identify a family member. We recruited participants using community-based recruitment routes including social media, and third sector organisations/nongovernment organisations (NGOs) who distributed project information and ‘expression of interest’ forms to their membership and networks (see Additional file 2). Inclusion criteria were as follows: we recruited participants who were aged ≥ 18 years, had had ≥ 1 stroke 3–60 months previously, were interested in learning how to cope with self-reported anxiety and/or depression, could speak and understand conversational English, and were not participating in another concurrent trial. Exclusion criteria were as follows: Stroke survivors who had attended an MBSR course in the last 3 years could not follow a two-stage command [24] and/or scored < 4 on each sub-scale of the Hospital Anxiety and Depression Scale (HADS) [25]. The latter criterion being the recommended cut-off for stroke survivors [26]. A project-specific Standard Operating Protocol (SOP), SOP 1, outlined procedures to be followed should an individual score ≥ 11 on HADS, indicating moderate to severe symptoms that may require further investigation, e.g. by a general practitioner. Note that a current prescription for anxiolytics or antidepressants was not an exclusion criterion. If screening identifiesd a potential participant who required GP referral, that individual may subsequently have been prescribed medication; this is a feature of the pilot work. In a future trial, where participants will be randomised to two arms and there is equal probability of people being on medication in each arm, it will not be an issue. SOP 2 described procedures to be followed should an individual express suicidal ideation.

We contacted people who had returned completed ‘expression of interest’ forms by telephone to answer questions and gauge eligibility. We asked stroke survivors to consider nominating a family member to take part with them. We then arranged to visit people at home and, once informed consent had been obtained, collected demographic data and administered the Montreal Cognitive Assessment (MoCA) [27] and the Behavioural Assessment of Dysexecutive Syndrome scale (BADS) [28]. We planned to use the latter to help determine whether there was a threshold at which HEADS: UP would constitute too great a burden for participants with communication and/or cognitive impairment. We gave nominated dyadic partners (family members) a family member-specific project information leaflet and consent form. Data were collected from family members only after we had obtained their informed consent.

### Data collection

Data were collected from stroke survivors and family members. Whilst the stroke survivor was the primary unit of analysis we were interested to see if in a definitive trial we should also be measuring family members’ outcomes. This decision reflected our interest in exploring whether dyadic participation might support stroke survivor adherence to the intervention in the short- and long-term and whether family members participating in a dyad might report improved mood and quality of life outcomes [18, 29]. We planned to collect quantitative data at three time points: baseline, Time 1 (within 2 weeks of completing the HEADS: UP course), and Time 2 (3 months later). We asked stroke survivors and family members to complete four self-report measures: Depression Anxiety Stress Scales (DASS) [30]; Beck Depression Inventory (BDI-II) [31]; Beck Anxiety Inventory (BAI) [32]; EuroQol-5 Dimensions-5 Levels (EQ-5D 5L) [33], and to record any personal mindfulness practice they engaged in outwith the weekly sessions, using a project-specific log. We asked stroke survivors to also complete the Stroke-Specific QOL (SS-QOL) [34] and the Stroke Impact Scale (SIS) [35]. We asked family members who self-identified as caregivers to complete the Carer Strain Index [36]. Prepaid addressed envelopes (PAE) were provided for the return of completed questionnaires. Any non-responders were offered other supported-completion options, i.e. over the phone or face-to-face.

At Time 1, we used focus groups to explore participants’ experiences of attending the HEADS: UP course, acceptability of content, materials and delivery, course participation and adherence (class attendance; personal practice). We also asked about acceptability of the HEADS: UP course in relation to managing symptoms of anxiety and depression. We used interviews with any participants unable to attend focus groups. We met with the trainers to record their reflections on delivering HEADS: UP, intervention fidelity and prospective 'models' for delivery and rollout. All focus groups and interviews were recorded and transcribed; we used field notes to capture non-verbal communication. Processes for data transfer and sharing complied with the requirements of the General Data Protection Regulation (2016) [37].

### Data analysis

We used descriptive statistics to summarise characteristics of participants, their outcomes and any adverse events, using SPSS statistical software; no formal significance tests were undertaken as non-randomised feasibility studies such as this are not powered to test for significance [38]. We analysed the qualitative data using a framework comprising domains of the Template for Intervention Description and Replication (TIDieR) [39] supplemented by inductive themes. In an iterative process, qualitative data was reviewed by two researchers (ML/BD) to identify key themes relating to feasibility and acceptability. We used the findings from both data sets to identify opportunities for optimising aspects of the HEADS: UP course and research processes (see Fig. 1).

## Results

As we were unable to collect data at Time 2, we present here baseline and Time 1 results only.

### Feasibility: research processes

#### Recruitment

**Fig. 2:**
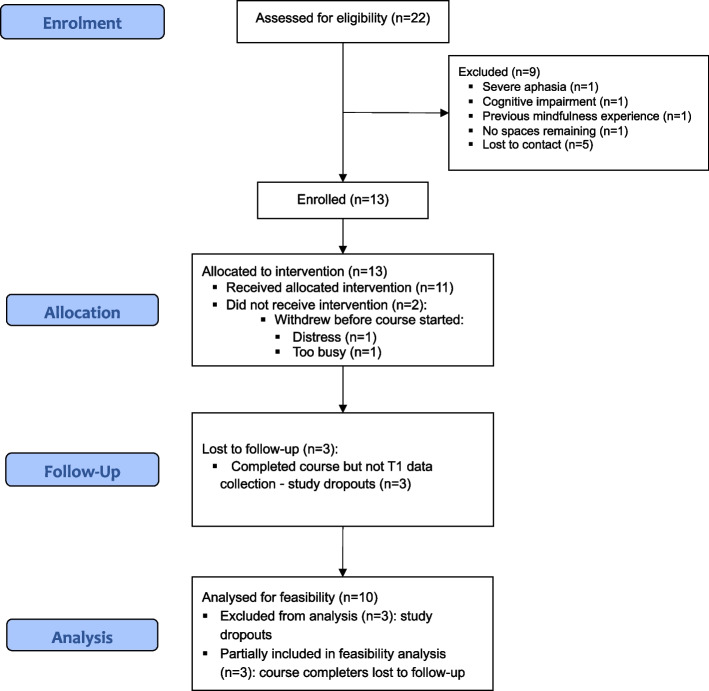
HEADS: UP Study 1 CONSORT flow diagram

We recruited over 9 weeks (August to October 2019). Thirty-one individuals expressed an interest; 22 were assessed for eligibility; 13 (59.1%; female *n* = 4, 30.8%) were enrolled (1.44 per week). Nine potential participants (40.9%) were excluded during screening (lost to contact *n* = 5, 55.6%; severe aphasia *n* = 1, 11.1%; severe cognitive impairment *n* = 1, 11.1%; previous MBSR experience *n* = 1, 11.1%; recruitment closed *n* = 1, 11.1%) (see Fig. 2 CONSORT flow diagram). We actioned SOP 1 (high HADS scores) for 12 (92%) of the 13 enrolled participants.

Five participants nominated a family member to take part with them (female *n* = 4, 80%; spouse *n* = 4, 80%; peer *n* = 1, 20%). Participants were aged 63.3 (standard deviation (SD) 10), male *n* = 9 (60%), 28 (interquartile range (IQR) 34) months post-stroke and educated to college level (or higher) *n* = 10 (76.9%). All were Caucasian. Family members were aged 67.7 (SD 9.2), male *n* = 1 (20%) and educated to college level (or higher) *n* = 2 (40%); all were Caucasian (see Table 1).

**Table 1:** Participant characteristics — Studies 1 and 2

	Study 1 (*n* = 13)		Study 2 (*n* = 9)	
	**N**	**%**	**N**	**%**
**Age** Median (IQR)	66 (14)		58 (12)	
**Gender**				
Male	9	69.2	4	44.4
Female	4	30.8	5	55.6
**Time post-stroke (months)**				
Median (IQR)	28 (34)		23 (10–38)	
**Living arrangements**				
With spouse/family	11	84.6	9	100
Alone	2	15.4	0	0.0
**Ethnicity**				
White Scottish	10	76.9	4	44.4
White Other British	2	15.4	3	33.3
White Non-Specific	1	7.7	2	22.2
**Employment status**				
Retired	8	61.5	3	33.3
Part-time employed	1	7.7	2	22.2
Full-time employed	2	15.4	0	0.0
Unemployed	0	0.0	2	22.2
Part-time volunteer	0	0.0	1	11.1
Long-term sick	2	15.4	1	11.1
**Highest educational attainment**				
Completed secondary	3	23.1	1	11.1
College or above	10	76.9	8	88.9
**HADS** mean (SD)				
Total	18.62 (4.9)		22.4 (6.7)	
HADS-A	10.08 (2.9)		11.4 (3.0)	
HADS-D	8.54 (4.3)		11.0 (4.4)	

*HADS* Hospital Anxiety and Depression Scale, *IQR* interquartile range, *SD* standard deviation

#### Completion of outcomes

Mean change in outcomes scores across the scales were positive and in the expected direction for both stroke survivors and family members (see Additional file 3). Data reporting completion of PROMs is presented in Table 2.

**Table 2:** Completion of PROMS — Studies 1 and 2

		Study 1		Study 2
		Stroke survivors (*n* = 13)	Family members (*n* = 5)	Stroke survivors (*n* = 9)
Baseline	At least partial completion of all PROMs	10 (76.9%)	3 (60%)	5 (55.6%)
	At least partial completion of ≥ 1 PROM	0 (0%)	1 (20%)	0 (0%)
	No completed PROMS	3 (23.1%)	1 (20%)	4 (44.4%)
Time 1	At least partial completion of all PROMs	6 (46.2%)	3 (60%)	5 (55.6%)
	At least partial completion of ≥ 1 PROM	1 (7.7%	0 (0%)	0 (0%)
	No completed PROMS	6 (46.2%)	2 (40%)	4 (44.4%)

*PROMS* Patient-reported outcome measures

### Retention: study completers and course completers

Seven (53.8%) participants were ‘study completers’, i.e. engaged in quantitative data collection at both time points. Six (46.2%) did not engage in quantitative data collection at Time 1 (bereavement (*n* = 1); time constraints (*n* = 1); distress (post-consent and pre-course; *n* = 1); lost to contact (*n* = 3)). None of the participants in dyadic partnerships were study dropouts. Nine (69.2%) of the 13 enrolled participants were ‘course completers’, i.e. had attended ≥ 4 of the 8 ‘core’ sessions; median attendance rate 7 sessions (IQR = 1). Of 3 participants who were study dropouts, all were ‘course adherents’ having attended 7 sessions (median; IQR = 1).

### Adverse events

No adverse events relating to the HEADS: UP course were recorded. The SOP 2 for suicidal ideation was not activated. One consented participant withdrew prior to course start citing ‘distress’.

### Qualitative results

At Time 1, we held two focus groups (FG1 *n* = 5; FG2 *n* = 5) and two phone interviews. We report the findings below using TIDieR domains 3–8 and inductive themes as subheadings. Key: Stroke survivor (SS) plus participant ID number, e.g. SS10; Focus Group (FG); Interview (Int).

### Feasibility and acceptability of research processes

#### Screening

Participants understood the importance and relevance of eligibility screening and described the process as ‘well thought out’. However, completing the MoCA/BADS was burdensome:

[the MoCA & BADS were] quite demanding ... mentally, it got a bit tiring, particularly towards the end of it … I did OK, but it really taxed me (SS10, Int2)

#### Completion of questionnaires

Participants often described an altruistic motivation for completing the questionnaires, but the number of questionnaires was overwhelming:

It does wear you [out] you know ... maybe it is a little bit too much, but I don’t know, I haven’t went [sic] through it all yet, I have done about 3 pages … (SS10, Int2)

One participant described their reluctance to report accurately the help they needed; another pointed out that for people with physical disabilities, completing paper forms was challenging. They welcomed a suggestion to make the questionnaires available online.

### Feasibility and acceptability of the HEADS: UP intervention

#### Course preparation

Participants had received pre-course illustrated information sheets that gave them an idea of what to expect from the course. Not ‘coming in cold’ was an important factor in alleviating anxiety about embarking on something new and unfamiliar:

The [accessible information sheets] were absolutely fabulous! I have never been given such clear instructions … (SS3, FG2)

In week 1, participants attended an introductory session ahead of the core course (weeks 2–9). This also proved helpful in reducing any anxiety about taking part in a group and the actual course:

[the introductory session] was quite useful because it did give us a chance to meet each other and hear the stories … [it] was a good way into what mindfulness was about because not everybody maybe knew about it (SS2, FG2)

#### Learning mindfulness techniques

Most participants found learning the various breathing meditations particularly helpful in controlling feelings of anxiety, as they were available to them whenever they needed them, irrespective of their immediate environment:

I think the breathing for me is really, really good … it works for me as well. Sometimes situations happen, and as I say especially with the breathing, you can do it any time and it works (SS6, FG1)

For some participants, other mindfulness techniques proved challenging. For example, several people found the body scan required some time to master, but once achieved, this could be used in the same versatile way as meditations of the breath:

I just couldn’t really get into it and I just I thought it was a waste of time to be honest ... what’s the point? Well, I find myself noo [now] on the bus doing it! I realise I am actually doing it and yet I didn’t like it [laughter] (SS7, FG2)

#### Personal practice

Participants received paper copies of weekly session material from the HEADS: UP manual along with audio recordings of the mindful practices they had been taught. Participants engaged well with these resources, making frequent use of them. As with other practices, they found them versatile and ‘portable’, drawing on them when and wherever they recognised rising symptoms of anxiety:

I find the voice files very, very helpful. I used it while I was sitting in the hospital with my wife, trying to comfort her. But it also helped me to reduce [my] anxiety (SS3, FG2)

Participants understood the importance of personal practice, essential to master the skills taught during the course, and in general found the supportive materials helpful:

It’s not a quick fix and you have got to persevere at it … it is practice, practice, practice and you have got to be prepared to do it or it is a waste of time coming [to the class] (SS6, FG1)

Finding the time and motivation to practice was often challenging, especially for participants who experienced fatigue, or found that busy lives intruded:

There doesn’t seem to be a great time then for me. It is either ‘I have got to walk the dog’ or ‘I have got to do this’, ‘I have got to shop’ or whatever. I don’t know. I found it difficult to fit it into the day and then in the night I am generally far too tired, and I fall asleep even if I mean to do it [the practice]. So, I found that a bit tricky you know, to find a good time to do it (SS11, FG1)

However, participants understood the importance of persevering with their mindfulness practice and incorporating it into everyday life, but many were unsure about how they could do this, once the course ended:

I am not sure how I will take it forward although I want to … (SS3, FG1)

#### Groupwork

Participants found being part of a group with common experiences and goals beneficial, growing in their mindfulness experience together:

it was good that I was learning new skills, I was listening to other participants and getting feedback … it is the first time I have ever been in a group with people that ... [are in] a similar position [have had a stroke] (SS7, FG2)

#### Dyads

Participants who attended in dyadic partnerships found working with a family member beneficial as their post-session discussions reinforced their learning from the weekly sessions and meant they could experience mindful moments together outwith the course. One participant, who had decided to attend alone, described belatedly understanding the benefit of attending with a family member having observed others’ interactions:

The fact that you have come [as a couple], and you can both hear it [mindfulness] fresh, and you know how to deal with it, … I think it probably works better than coming here on your own … and I can’t believe I have just said that! (SS3, FG1)

#### Personal impacts

Participants described being ‘at the beginning’ of a long journey but reported perceived benefits including stress reduction, ‘living in the moment’, and improved relationships:

I think I am a different person … I don’t [get angry with] my husband as much now, so I think he is quite happy [laughter in the group]. I am a much calmer person … (SS3, FG1)

### Optimisation

Optimisation of the intervention and research processes was undertaken following the completion of data analysis. However, imposition of Covid-19 restrictions which had resulted in early termination of Study 1 necessitated changes to the original design of the planned programme of work. Therefore, taking a pragmatic approach, and in collaboration with the funder, we elected to use this restriction on in-person meetings as an opportunity to explore an alternative delivery medium, acceptable within contemporaneous Covid-19 restrictions. Consequently, we decided to adapt the optimised, in-person intervention and research processes for online delivery and to test these in a subsequent non-randomised feasibility study (Study 2). Table 3 presents an overview of the optimisation and adaption processes (both studies).

**Table 3:** Optimisation and adaptation processes

Item		Evidence /identified need for change	Optimisation (post-Study 1)	Adaptation (pre-Study 2)
Research processes				
Eligibility criteria	Time post-stroke	Expressions of interest from participants 3–60 months post-stroke	Removed upper limit from time post-stroke	No further change made
	Participation in other research studies		Removed restriction (except for other mood disorder studies)	No further change made
	Internet access	Covid restriction imposing ‘social distancing’	Not applicable	Added requirement to be able to access the internet, preferably using a desktop, laptop or tablet
Recruitment of Dyads	To partner stroke survivor participant	Observation and reports of differing modes of participation across dyads	Participant information amended to clarify that a FM identified by them at enrolment was not required to attend the weekly sessions	Advised participants that a FM identified by the participating stroke survivor at enrolment may have a role assisting the stroke survivor to engage with the online platform, course materials, and/or personal practice outwith the sessions
Recruitment	Network contacts	Changes in organisational structure resulted in loss of established contacts	Implemented strategies to improve communications, including in-person meetings	Continue to work to improve communications; arrange online meetings
	Social media	Stated aim to develop additional routes to recruitment to maximise potential of the community-based recruitment strategy	Established HEADS: UP Twitter account	Identified and targeted influencers in stroke research
	Mapping routes to recruitment	Lack of data	Added the question ‘How did you hear about HEADS: UP research?’ to the screening process	Add question ‘How did you hear about HEADS: UP research?’ to web contact form
	Lack of diversity in participant profile	Participant characteristic data (white; young; educated; employed)	Developed strategies to extend recruitment to a wider audience	Moved recruitment processes online which extended recruitment catchment area to the whole of the UK
Screening process	Screening checklist/script	Researchers and potential participants reported finding the process time consuming and burdensome	Streamlined: screening undertaken using a researcher ‘script’ during a single meeting (Zoom or phone); with ineligibility cut off points signposted throughout as appropriate. Eligible individuals were then sent information sheets and consent form by post/email according to preference; electronic signatures accepted	Text added to reflect online nature of the research; logistics questions added about internet access, IT skills and need for assistance
			Some participants found returning email attachments challenging	Two statements sent in a single email: ‘I consent…’ and ‘I do not consent…’. Participants selected the statement they considered appropriate and copied it into their email reply
		Researcher and potential participants found some HADS questions inappropriate for stroke survivors	Removed HADS; replaced with PHQ-4	Trialled successfully
	Screening assessment tools	Participant burden (as above)	Removed BADS; retained the two-stage command	Replaced MoCA with MoCA Blind
Quantitative data collection	Outcomes measures	Partial completion	Changed order of outcomes measures to favour most likely candidate	Uploaded to REDcap, online survey software; forced response option deployed
			Planned to introduce text prompts to remind participants to complete forms	Used the Janet, a 1-way web-based text-messaging service, to send text prompts reminding participants to complete forms
		Reported participant burden (multiple measures)	Planned to trial use of online data collection system e.g. REDCap, a secure web application for building and managing online surveys; successfully trialled at Time 1	Uploaded to REDCap; simple feedback form to assess ease of completion inserted after every measure
		Missing data due to unreliability of securing postal returns (mobility; size of package; postal worker strikes)	Planned to trial use of online data collection system e.g. REDCap; successfully trialled at Time 1	Uploaded to REDCap (postal returns for participants expressing a preference for paper copies)
		Personal Practice Logs (PPLs) Participant reports that completion instructions were unclear, which led to over-completion, which was burdensome	Form format simplified; instructions revised	PPL adapted for upload to REDCap, with forced response option deployed
HEADS: UP intervention				
Manual	Distribution	Expressed concerns (e.g. people with cognitive impairments) regarding need to prepare for sessions?	Send weekly session chapter as email attachment; made weekly session chapters available in alternative information repositories/ communication platforms i.e. Blackboard, Padlet	Whole manual provided electronically following completion of the Introductory session of the course; also made available on Padlet. Paper copies posted, if required
		Expressed concerns over perceived need to read course material ahead of sessions	Reinforced in emails etc. that reading the manual is not a pre-course/pre-session requirement	Made available in Padlet
		Reported participant burden	Send paper/ e-copy (according to preference) at course end	As above
			Reinforced messaging that reading the manual is not a requirement/essential	Messaging repeated across information repositories /communication platforms
Trainers	Train the Trainer (TtT) training	Trainers reported receiving questions from participants about the research processes, which they struggled to answer	TtT material extended to cover research processes in more detail	TtT material adapted to reflect online delivery; IT session added (supported by online access support materials/tuition)
	Trainers’ materials/ templates	Trainers reported receiving questions from participants about the research processes, which they struggled to answer	Amended weekly email templates to remind participants to contact the research team with any research process questions	No further change made
The 9-week course	Course length	Participants reported feeling that going ‘it alone’ after the 9-week course would be challenging	Planned to introduce a one-off follow-up session at 6 weeks post-intervention	One-off follow-up online session planned at 6 weeks post-intervention
		Trainers reported session 4 was ‘too busy’; they struggled to include all the session material	TtT: busy-ness of week 4 highlighted; trainers encouraged to maintain fidelity to course structure	Fidelity checklist developed with ease-of-use in mind

### Study 2: HEADS: UP online delivery

In Study 2, we tested feasibility and acceptability of the HEADS: UP Online intervention and research processes in a non-randomised study, aiming to answer the question: what elements of design, delivery and study processes require to be optimised ahead of a HEADS: UP Online RCT?

The structure of the intervention was unchanged from Study 1. It was delivered online, via the communication platform, Zoom^©^. Changes to course materials and their delivery included providing the manual in its entirety in week 1 (paper/electronic, according to preference), and uploading audio resources to HEADS: UP’s online Padlet^©^ noticeboard after each weekly session. HEADS: UP Online was delivered by only one experienced MBSR trainer. The trainer had delivered HEADS UP in-person in study 1, having previously undertaken TtT training. As before, the trainer recorded divergence from session plans and provided feedback to enhance future delivery.

We recruited using a continually expanding community-based strategy, e.g. professional and other networks, dedicated stroke/rehabilitation Facebook groups, and Twitter. A novel feature of this was the use of short ‘recruitment’ videos made by stroke survivors and family members who had participated in Study 1 [40]. We aimed to recruit 10 stroke survivors. Inclusion/exclusion criteria were broadly similar; however, to reduce participant burden, we replaced HADS with the Patient Health Questionnaire-4 (PHQ-4) [ [41] and MoCA [27] and BADS with the Modified Telephone Interview for Cognitive Status (TICS-M) [42].

### Data collection

Within the limitations of our funding, we had only a short amount of time in which to conduct this second non-randomised study; consequently, we collected data at baseline and at Time 1 (i.e. within 2 weeks of course completion) only. Quantitative data (i.e. DASS, BDI-II, BAI, EQ-5D 5L, SIS, and a bespoke personal practice log) were collected using REDcap^©^ survey software. Data were not collected from family members; the stroke survivor was the primary unit of analysis. At Time 1, we used Zoom recording (video and audio) and transcription functions to collect qualitative data (focus groups and interviews).

### Data analysis

Quantitative and qualitative data were analysed and reported as for Study 1. We used the findings from both data sets to identify opportunities for optimising the online research processes and course (see Fig. 1).

## Results

### Feasibility: research processes

#### Recruitment

**Fig. 3:**
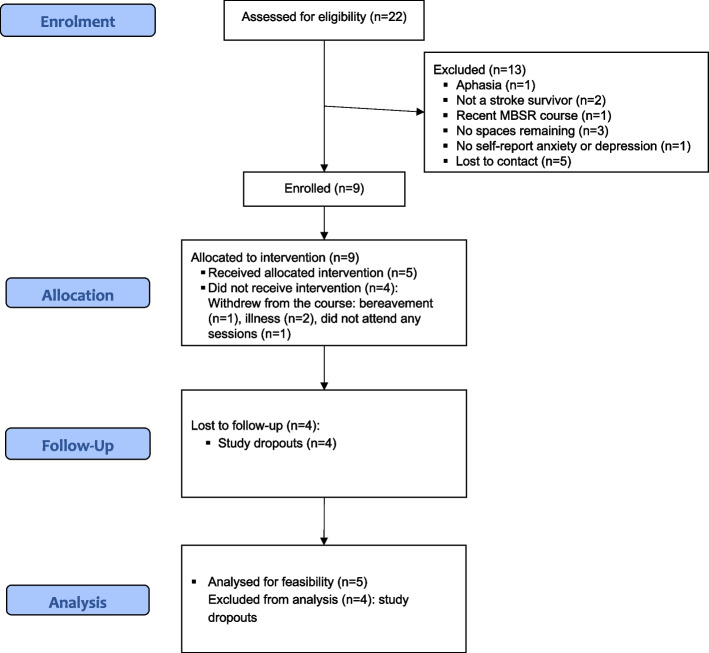
HEADS: UP Study 2 CONSORT flow diagram

We recruited over 7 weeks (October to December 2020). We made project information widely available through recruitment networks (see Additional file 2), highlighting the need for people to contact the research team if they were interested in the research. Twenty-five individuals made contact, including three third-party enquires. Twenty-two potential participants were assessed for eligibility; nine stroke survivors were enrolled (1.29 per week). Thirteen potential participants (59.1%) were excluded during screening (lost to contact *n* = 5, 22.7%; not stroke *n* = 2, 9.1%; severe aphasia *n* = 1, 4.5%; previous MBSR experience *n* = 1, 4.5%; not anxious/depressed *n* = 1, 4.5%; recruitment closed *n* = 3, 13.7%) (see Fig. 3 Study 2 CONSORT diagram). We applied the SOP for individuals scoring ≥ 11 on HADS for all enrolled stroke survivors.

Three participants nominated a family member to take part with them (female *n* = 3, 100%; spouse *n* = 2, 66.7%; parent *n* = 1, 33.3%). Stroke survivors were aged 54.8 (SD 12.7), male *n* = 4 (44.4%), 23 (IQR 28) months post-stroke and educated to college level (or higher) *n* = 8 (88.9%); all were Caucasian; one (11.1%) had mild/moderate expressive aphasia (see Table 1).

#### Completion of outcomes

At baseline, five (55.6%) participants at least partially completed each PROM. Reasons for non-completion included Covid-19-related ill-health (*n* = 2, 22.2%), bereavement (*n* = 1, 11.1%) and non-attendance at sessions (*n* = 1). At Time 1, all participants reported completing the PROMs; however, five data sets were lost in the post due to postal strikes that coincided with Time 1 data collection, resulting in receipt of only the five sets of PROMs completed online. Six (66.6%) participants completed practice logs; *n* = 5 returned ≥ 7. The mean change scores across PROMs were positive and in the expected direction (see Additional file 4).

#### Retention

Five (55.6%) participants were ‘study completers’. Four (44.4%) did not return quantitative data at Time 1 although all nine reported having returned completed PROMs. Two of the three participants in dyadic partnerships were study dropouts. Six (66.7%) participants were ‘course completers’; mean attendance rate was 5.1 sessions.

#### Adverse events

No adverse event in relation to the HEADS: UP Online course was recorded; SOP 2 (suicidal ideation) was not activated.

### Qualitative results

At Time 1, we conducted two focus groups online (*n* = 6; three participants per group). We report the findings below.

### Feasibility and acceptability of research processes

#### Recording personal practice

Some participants found completing the weekly practice log a daunting and tiring task. However, with additional guidance, they were able to use it as intended, i.e. as a low-burden data collection tool, and some found it a useful tool for prompting reflection on perceived progress:

The personal logs … actually helped because I suddenly thought – you know, I am actually reflecting on how far I have come (SS3)

### Feasibility and acceptability of the HEADS: UP intervention

#### Attending HEADS: UP online

Initially, participants approached the online course with caution — they were uncertain about meeting others on Zoom, and whether mindfulness could work when delivered ‘remotely’. However, as the course progressed, participants became aware that they were developing a connection or bond with fellow group members:

I really do feel that I am part of the group, I hope we get the opportunity to actually meet each other [in person] (SS9)

#### Impact of learning mindfulness

Learning mindfulness impacted daily life. Participants found their newly acquired skills were helping them feel that they were coping with stressors more effectively. This included managing responses to stressful situations and improvements in the quality and nature of interpersonal relationships:

15 minutes of ‘loving kindness’ and I am just a different person; I can deal with what I need to deal with much better (SS9)

If somebody upsets me, I will take a mindful breath and deal with that (SS5).

#### Intention to continue practising

Acknowledging the impacts such as those described above prompted participants to declare that they would continue to practice mindfulness once the course ended:

It’s definitely something that I am going to put into practice and keep doing in my life because it’s made a huge difference to how I feel [psychologically] (SS6)

[Doing the HEADS: UP course] helped dramatically; I can’t explain how much it has helped. I feel completely different (SS3)

### Optimisation

Optimisation of the online intervention and research processes was undertaken following completion of Study 2 data analysis (see Fig. 1). Table 3 presents an overview of optimisation and adaption processes (both studies).

## Discussion

HEADS: UP, whether delivered in-person or online, was found to be feasible and acceptable to stroke survivors.

### Feasibility and acceptability of research methods, materials and processes

#### Recruitment

We used a community-based recruitment strategy which proved exceptionally effective as we met our recruitment targets in a matter of weeks. A review of recruitment to stroke rehabilitation interventions [43] found a median monthly recruitment rate of 1.5 (median; IQR 0.71–3.22); rates exceeded in the two studies reported here. However, the recruitment targets were small, and the strategy has yet to be tested in definitive trial.

Across both studies, participants were, in broad terms, white, young (when compared with the average age of UK stroke survivors, i.e. 77 years median; IQR 67–85 [44]) and well-educated. Mind–body practices such as yoga and mindfulness typically attract younger, well-educated adults, predominantly female [15, 45, 46]. In Study 2, in which we used only online recruitment methods, we extended the recruitment catchment area to include participants from across the UK; however, as people from diverse ethnic minority communities and from areas of socioeconomic deprivation were not adequately represented in our sample, in future work, in collaboration with equality and diversity specialists and relevant NGOs, we will further develop the recruitment strategy to ensure greater representativeness.

Participants and researchers reported finding Study 1 screening and enrolment processes lengthy and cumbersome; optimisation and adaptation rendered them feasible and acceptable when tested online. Similarly, Beauchamp and colleagues (2023) [47] found a lengthy telephone questionnaire may have influenced rates of withdrawal or loss to follow-up.

#### Data completion

In Study 1, HEADS: UP participants encountered several factors that hindered completion and return of paper-based PROMS and practice logs. As a result, we optimised quantitative data collection methods by reducing participant burden, and switching to an online system (REDcap), which resulted in greatly improved completion rates and participant satisfaction (see Table 3). Similarly, in a feasibility and acceptability study of mindfulness-based interventions and relaxation, the 50% return rate of completed participant diaries prompted the researchers to suggest that self-report diaries may not be the most effective means of tracking participants’ personal practice [48]. However, in a feasibility and acceptability study of post-stroke physical activity, findings were more ambiguous as whilst some participants found the task of competing diaries ‘overwhelming’, most considered using the diaries to record and reflect on progress to be motivational [49]. Additionally, participant feedback indicated that a short, simple format was preferred, along with an option for electronic completion [49]. However, in Study 2, postal return of PROMS continued to be problematic as temporary closure of the local post office and a postal strike coincided with Time 1 data collection, and whilst all nine participants confirmed verbally that they had returned completed PROMS, only five packs were received. In future stages of the research, we will encourage and support online completion of PROMS to try to reduce dependence on the safe return of paper copies.

### Feasibility and acceptability of HEADS: UP (course and materials)

#### The group experience

Course adherence was good in both studies, irrespective of whether a participant was a study dropout. Overwhelmingly, learning and practising in a group with others with similar/relatable experience, in-person or online, was an aspect of the intervention that strongly supported course adherence. This is a feature common across stroke rehabilitation studies in which feeling supported is dependent on the group approach and the benefits that accrue from shared experiences [50]. Research participants report finding learning in a group to be supportive and motivational [50–52], particularly when observing others’ successful achievements [50, 52]. This is an important consideration for a definitive RCT control condition, where a structurally equivalent control 'intervention' will be desirable [53].

#### Dyads

Prior to study commencement, we hypothesised that participating in HEADS: UP as a member of a dyad would enhance course engagement and adherence and ultimately impact long-term therapeutic benefit [52]. Studies of other ‘survivor’ populations have reported a range of benefits gained from dyadic support, including skill acquisition and increased confidence and self-efficacy [54–56]. In the event, there were few dyads across both studies. During screening, some stroke survivors expressed a preference for attending without a family member, as engaging in the intervention unaccompanied constituted ‘alone time’. Others felt that the potentially sensitive or emotionally charged nature of their engagement might be inappropriate to share in the presence of a family member (typically a spouse or child), reflecting findings from earlier studies [e.g. 51]. Those who did take part in a dyad found the opportunity for (mutual) support helpful and participating in a dyad did not impact adherence; however, given the small number of dyadic participants, further exploration is warranted before firm conclusions may be drawn.

#### Course materials

Pre-course materials designed to allay participants’ fears and anxieties about the course and being in a group with strangers were well received. However, the response to the course manual was less positive, with participants finding the paper version daunting due to its large size. Double spacing and a large sans serif font had resulted in a 200-page manual. Despite using a variety of media and formats (e.g. email, large font wording on the cover, verbal reinforcement), we found it challenging to convey to participants that the manual was not required reading. Rather, it was intended as a supplement to the course or as support material, particularly for those with cognitive impairments who wanted to feel prepared ahead of weekly sessions. Tailoring interventions and accompanying materials and processes to the needs of an individual is a feature of stroke self-management rehabilitation interventions [e.g. 50, 57] and will be explored in future HEADS: UP research. Findings from an RCT testing a meditation intervention with stroke survivors and caregivers indicated that screening could be used to identify potential technology-related challenges, technical support requirements, and individuals’ preferences with regard to communication, including reminders (e.g. short-message services) ahead of study commencement [47].

#### Course content (practices)

Responses to the different practices were varied, with many participants cherry-picking those they found most accessible, rather than adhering to the assigned practices for that week. This is a finding reflected in other mindfulness intervention studies where participants reported selecting the practices they found most accessible and acceptable [e.g. 48, 58]. Overall, participants found HEADS: UP acceptable and beneficial, and expressed desire and intention to continue to practice long-term. Similarly, Wang and colleagues [48] found that even with low practice frequency, most participants reported finding mindfulness and relaxation practices beneficial, and expressed intentions to maintain future practice. However, participants in the HEADS: UP study also identified barriers to future practice which included competing priorities, busyness and cognitive impairments, including memory and concentration. Many participants anticipated that initiating and embedding this new habit into daily life would be challenging; a finding echoed elsewhere [e.g. 48], highlighting behaviour change (initiation and maintenance) as an important area upon which to further focus our attention in future work [59].

#### Benefits and harms

Whilst non-randomised feasibility studies are not designed to test intervention effectiveness, we were interested to learn whether participants perceived that they had benefited from or been harmed in any way by participation in the HEADS: UP course. No harms were attributed to HEADS: UP participation, but perceived benefits were described by some participants. These included reduced reactivity to stressful situations, a greater sense of calm and improved interpersonal relationships, benefits also observed by Parkinson et al. (2023) [58] in their study of care partners engaging with an online mindfulness course.

#### Practising mindfulness in the long-term

In terms of future practice, participants acknowledged that acquiring mastery of mindfulness skills required long-term commitment to regular practice, and they were uncertain as to how best to manage that once the support available to them during the HEADS: UP research was withdrawn. Our understanding of behaviour change [60] and self-management elements of this research had informed the theoretical underpinning from the outset. For example, in our development work [18], we mapped behaviour change techniques [61] embedded in the course manual and used this information to further enhance the manual (i.e. insert additional BCTs to foster behaviour change i.e. initiation and maintenance of a new behaviour, mindful practice and to support self-management efforts). In recent feasibility studies of MBIs for stroke survivors, technological and non-technological options for providing tailored prompts and cues (e.g. audible reminders/alarms, short text messages, strategically placed Post-it^©^ notes) were identified by participants as essential to support behaviour change and eventual development of a mindfulness habit [57, 62]. The learning derived from the two feasibility studies reported here, and from other relevant research, evidence will inform future HEADS: UP research.

## Limitations

Across the two studies, participants could be characterised as young (for an adult stroke population), white and well-educated. Extending the recruitment catchment area to the whole of the UK in Study 2 did not result in greater ethnic diversity in the participant group. Consequently, in future research, we will work with organisations dedicated to improving equity of access across diverse socioeconomic and minority ethnic populations to develop a more inclusive recruitment strategy.

Imposition of Covid-19 restrictions in 2020 resulted in the sudden cessation of in-person research, which required a major alteration to the design of the planned programme of work. However, we maximised this unlooked for opportunity by taking a pragmatic approach and redesigning the study to allow us to deliver the study objectives namely completion of feasibility and acceptability testing of HEADS: UP. Notably, the sudden cessation of Study 1 coincided with planned collection of 3-month follow-up data, and in Study 2, time and budget restrictions resulted in collection of baseline and post-intervention data only. We acknowledge this as a limitation of the research, being aware that lack of long-term follow-up is a common limitation of stroke rehabilitation intervention studies [e.g. 63]. In a future HEADS: UP RCT, we will work to understand if participants are able to continue to practice and develop mindfulness skills in the long-term and how this may impact anxiety and depression outcomes.

## Conclusions

Stroke survivors found a stroke-specific psychological self-management intervention, HEADS: UP, feasible and acceptable, whether delivered in-person or online. This important feasibility and acceptability work, which incorporated iterative rounds of optimisation and adaptation, found that it is feasible and acceptable to further test HEADS: UP with stroke survivors in a subsequent pilot RCT.

## Ethical approval

Study 1: Ethical approval granted: HLS/PSWAHS/18/196 *HEADS: UP.*

Study 2: Ethical approval granted: HLS/NCH/19/061 *HEADS: UP (Helping Ease Anxiety and Depression following Stroke) Online psychological self-management intervention: feasibility trial.*

## Supplementary Information

Additional file 1. Completed CONSORT checklist.

Additional file 2. Recruitment strategy.

Additional file 3. Study 1 PROMS data.

Additional file 4. Study 2 PROMS data.

## Data Availability

All quantitative PROMS data generated or analysed during this study are included in this published article and its supplementary information files. The qualitative datasets generated and analysed during both studies are not publicly available because, in line with contemporaneous conventions and university policies, participants in the HEADS: UP non-randomised feasibility studies gave consent for us to store their data for 5 years, after which time the data will be securely destroyed (in March 2025 for Study 1, and June 2026 for Study 2).

## Abbreviations

BADS: Behavioural Assessment of Dysexecutive Syndrome scale
BAI: Beck Anxiety Inventory
BD: Bridget Davis (second author)
BDI-II: Beck Depression Inventory
CD: Compact disc
DASS: Depression Anxiety Stress Scales
DIGS: Diagnostic Interview for Genetic Studies
EDI: Equality, Diversity, and Inclusion
EQ-5D 5L: EuroQol-5 Dimensions-5 Levels
FG: Focus groups
FM: Family member
HADS: Hospital Anxiety and Depression Scale
Int: Interview
IQR: Interquartile range
MBSR: Mindfulness-Based Stress Reduction
ML: Maggie Lawrence (first author)
MoCA: Montreal Cognitive Assessment
PAE: Prepaid addressed envelopes
PHQ-4: Patient Health Questionnaire-4
PROMs: Patient-reported outcomes measures
RCT: Randomised controlled trial
SD: Standard deviation
SIS: Stroke Impact Scale
SOP: Standard Operating Protocol
SS: Stroke survivors
SS-QoL: Stroke-Specific QOL
TICS-M: Modified Telephone Interview for Cognitive Status
TIDieR: Template for Intervention Description and Replication
TtT: Train the Trainer
UK: United Kingdom
