# Supplementary material for: In-person and online mixed method non-randomised studies exploring feasibility and acceptability of HEADS: UP, an adapted Mindfulness-Based Stress Reduction programme for stroke survivors experiencing symptoms of anxiety and depression
Source: Pilot Feasibility Stud. 2024 Sep 12;10:119. doi: 10.1186/s40814-024-01545-w (PMC11391595; doi:10.1186/s40814-024-01545-w)
Supplement: Supplementary file 2 — Additional file 2. Recruitment strategy. [file 40814_2024_1545_MOESM2_ESM.pdf]

Additional File 2 Recruitment Strategy

Draft Recruitment strategy

Aim

To develop a robust and enduring community-based recruitment strategy.

Actions

Personnel

- Recruit a researcher with time dedicated to this role (front-loaded)

Project contacts

- Set up a generic project email account (relevant team members can access)
- Set up a generic project phone number (relevant team members can access)
- Organise monitoring of these ((relevant team members)

Webpage

- develop a webpage for the project, including a webpage contact form
- Update regularly

Set up a database of network contacts (individuals and organisations)

- Name of contact person (with permission)
- Contact details, including website, if applicable (with permission)
- Any special instructions/comments, including preferred method of contact
- Actions with dates

Develop links with community organisations

- Identify relevant target organisations, community groups, media representatives (e.g. radio stations, local newspapers), social media groups (e.g. Facebook), etc
- Interact to establish a rapport/working relationship (in-person visits, phone calls, emails) and identify a key contact person
- Add to database (with permission)

Contact using preferred contact method

- Confirmation and summary of verbal /in-person meetings
- Informal updates at pre-specified times (set up calendar alerts)
- Formal update and progress reports at pre-specified times (set up calendar alerts)

Written materials

- Develop in collaboration with PPI (patient and public involvement) members

- Accessible (see accessible information guidelines)
- Plain English
- Illustrate (photos, graphics, etc)

Photographs, illustrations, and graphics

- Take photos to use in social media, presentations reports, etc (ethical approval required)

Equality, Diversity and Inclusivity

- Identify EDI organisations and sources of support e.g. Centre for Ethnic Research, DifferentStrokes (provide services for marginalised groups), Poverty Alliance

Social media presence

- Establish and continually expand networks (individuals, organisations, professional)
- Develop content (consider equality, diversity and inclusion) and post on a regular basis
- Post conference papers and other publications

Recruitment videos

Work with past participants to develop short videos (ethical approval required) to post on social media, use in recruitment meetings e.g.

- What's it like taking part in research (addressing potential anxieties)
- Experiences of taking part in research
- What's required of a research participant
