# Supplementary material for: In-person and online mixed method non-randomised studies exploring feasibility and acceptability of HEADS: UP, an adapted Mindfulness-Based Stress Reduction programme for stroke survivors experiencing symptoms of anxiety and depression
Source: Pilot Feasibility Stud. 2024 Sep 12;10:119. doi: 10.1186/s40814-024-01545-w (PMC11391595; doi:10.1186/s40814-024-01545-w)
Supplement: Supplementary file 3 — Additional file 3. Study 1 PROMS data. [file 40814_2024_1545_MOESM3_ESM.pdf]

Additional File Study 1 PROMS data

Table 4.1: Change in stroke survivors PROMs scores

	T0		T1		CHANGE
	**n**	**M (SD)**	**n**	**M (SD)**	**M (|%|)**
**HADS**					
TOTAL	13	18.62 (4.9)	7	10.14 (5.4)	-8.48 (45.5%)
HADS-A	13	10.08 (2.9)	7	6.43 (3.0)	-3.65 (36.2%)
HADS-D	13	8.54 (4.3)	7	3.71 (2.7)	-4.83 (56.6%)
**BAI**	9	9.89 (8.6)	5	7.20 (5.2)	-2.69 (27.2%)
**BDI**	9	20.67 (11.0)	5	9.8 (7.7)	-10.87 (52.6%)
**DASS**					
TOTAL	7	34.57 (27.8)	6	22.33 (15.6)	-12.24 (35.4%)
DASS-A	8	10.25 (8.5)	6	5.00 (4.1)	-5.25 (51.2%)
DASS-D	8	14.50 (12.5)	7	6.00 (6.1)	-8.50 (58.6%)
DASS-S	10	17.40 (12.0)	7	10.00 (6.0)	-7.40 (42.6%)
**EQ5D5L**					
INDEX	10	0.761 (0.10)	6	0.770 (0.08)	+0.009 (0.01%)
VAS	10	71.9 (16.65)	6	83.2 (11.78)	+11.3 (15.7%)
**SSQoL**					
SUMMARY	9	3.88 (0.67)	5	4.18 (0.72)	+0.30 (0.07%)
ENERGY	9	3.74 (1.42)	7	4.14 (1.05)	+0.40 (10.7%)
FAMILY ROLE	9	3.37 (1.12)	7	4.19 (0.92)	+0.82 (24.3%)
LANGUAGE	9	4.71 (0.33)	7	4.49 (0.69)	-0.22 (4.7%)
MOBILITY	9	4.83 (0.24)	7	4.60 (0.43)	-0.23 (4.8%)
MOOD	9	3.20 (1.08)	6	4.20 (0.62)	+1.00 (31.3%)
PERSONALITY	9	2.78 (1.67)	7	3.95 (0.85)	+1.17 (42.1%)
SELF-CARE	10	4.80 (0.46)	7	4.54 (0.82)	-0.26 (5.4%)
SOCIAL ROLE	9	3.02 (1.28)	6	3.73 (1.02)	+0.71 (23.5%)
THINKING	10	3.43 (1.12)	7	3.95 (1.04)	+0.52 (15.2%)
UPPER EXTREMITY FUNCTION	10	4.26 (0.82)	7	3.97 (1.21)	-0.29 (6.8%)
VISION	10	4.97 (1.23)	7	5.00 (0.00)	+0.03 (0.6%)
WORK & PRODUCTIVITY	10	3.47 (1.23)	7	4.00 (1.05)	+0.53 (15.3%)
**SIS**					
VAS	8	69.38 (25.63)	7	87.57 (4.86)	+18.19 (26.2%)
PHYSICAL	8	16.75 (3.54)	5	16.20 (2.86)	-0.55 (3.3%)
MEMORY	10	28.20 (6.18)	7	31.86 (2.67)	+3.66 (13.0%)
MOOD	8	33.25 (7.21)	6	38.33 (5.35)	+5.08 (15.3%)
COMMUNICATION	9	32.33 (3.43)	6	33.00 (1.79)	+0.67 (2.1%)
ADL	7	45.86 (3.98)	6	44.33 (5.68)	-1.53 (3.3%)
MOBILITY	10	42.50 (2.64)	6	42.00 (2.97)	-0.5 (1.2%)
HAND FUNCTION	8	18.75 (7.72)	5	21.60 (5.98)	+2.85 (15.2%)
MEANINGFUL ACTIVITIES	7	31.43 (6.48)	6	32.67 (7.89)	+1.24 (3.9%)
