# Supplementary material for: In-person and online mixed method non-randomised studies exploring feasibility and acceptability of HEADS: UP, an adapted Mindfulness-Based Stress Reduction programme for stroke survivors experiencing symptoms of anxiety and depression
Source: Pilot Feasibility Stud. 2024 Sep 12;10:119. doi: 10.1186/s40814-024-01545-w (PMC11391595; doi:10.1186/s40814-024-01545-w)
Supplement: Supplementary file 4 — Additional file 4. Study 2 PROMS data. [file 40814_2024_1545_MOESM4_ESM.pdf]

Additional File Study 2 PROMS data

Table 4.1: Change in SS OM Scores

	T0 n	T0 M (SD)	T1 n	T1 M (SD)	CHANGE M (|%|)
**BDI**	5	19.4 (8.2)	5	3.2 (3.3)	-16.2 (83.5%)
**BAI**	5	22.8 (11.8)	5	5.4 (5.3)	-17.4 (76.3%)
**DASS**					
TOTAL	5	46.8 (31.5)	5	6.4 (7.4)	-40.4 (86.3%)
DASS-A	5	9.2 (8.9)	5	0.8 (1.1)	-8.4 (91.3%)
DASS-D	5	19.2 (11.6)	5	1.6 (3.6)	-17.6 (91.6%)
DASS-S	5	18.4 (12.3)	5	4.0 (4.0)	-14.4 (78.3%)
**EQ5D5L**					
MOBILITY	5	2.2 (0.8)	5	1.8 (1.1)	-0.4 (18.2%)
SELF-CARE	5	1.6 (0.9)	5	1.2 (0.4)	-0.4 (25%)
USUAL ACTIVITIES	5	2.2 (0.8)	5	1.4 (0.5)	-0.8 (36.4%)
PAIN	5	2.6 (1.1)	5	2.0 (1.0)	-0.6 (23.1%)
ANX/DEP	5	2.8 (0.8)	5	1.4 (0.5)	-1.4 (50%)
VAS	5	53.6 (18.8)	5	81.0 (13.9)	+27.4 (51.1%)
INDEX	5	0.562 (0.235)	5	0.804 (0.193)	+0.242 (43.1%)
**SF-SIS**					
STRENGTH	4	4.5 (0.6)	5	4.0 (1.0)	-0.5 (11.1%)
MEMORY	5	3.6 (1.5)	5	3.2 (1.6)	-0.4 (11.1%)
EMOTION	5	3.2 (1.3)	5	4.8 (0.5)	+1.6 (50%)
COMMUNICATION	5	4.0 (1.0)	5	4.4 (0.9)	+0.4 (10%)
ADL	5	4.0 (1.0)	5	4.2 (0.8)	+0.2 (5%)
MOBILITY	5	3.6 (1.1)	5	4.4 (0.9)	+0.8 (22.2%)
HAND FUNCTION	5	4.0 (1.2)	5	4.2 (0.8)	+0.2 (5%)
SOC. PART.	5	2.6 (0.9)	5	3.8 (1.6)	+1.2 (46.2%)
INDEX	4	64.8 (24.9)	5	78.1 (23.7)	+13.3 (20.5%)
